# Transient Canonical Wnt Stimulation Enriches Human Bone Marrow Mononuclear Cell Isolates for Osteoprogenitors

**DOI:** 10.1002/stem.2241

**Published:** 2015-11-17

**Authors:** Agnieszka A. Janeczek, Rahul S. Tare, Edoardo Scarpa, Ines Moreno‐Jimenez, Caroline A. Rowland, Dominic Jenner, Tracey A. Newman, Richard O. C. Oreffo, Nicholas D. Evans

**Affiliations:** ^1^ Centre for Human Development, Stem Cells and RegenerationUniversity of SouthamptonSouthamptonUnited Kingdom; ^2^ Bone and Joint Research Group, Human Development and Health Academic Unit, Institute for Developmental Sciences, Faculty of MedicineUniversity of SouthamptonSouthamptonUnited Kingdom; ^3^ Institute for Life SciencesUniversity of SouthamptonSouthamptonUnited Kingdom; ^4^ Microbiology group, Chemical, Biological and Radiological DivisionSalisburyUnited Kingdom; ^5^ Clinical and Experimental Sciences, Faculty of Medicine, Institute for Life SciencesUniversity of SouthamptonSouthamptonUnited Kingdom

**Keywords:** Wnt signaling, Marrow stromal cells/mesenchymal stem cells, Osteoprogenitors, Stem cells, Fracture healing

## Abstract

Activation of the canonical Wnt signaling pathway is an attractive anabolic therapeutic strategy for bone. Emerging data suggest that activation of the Wnt signaling pathway promotes bone mineral accrual in osteoporotic patients. The effect of Wnt stimulation in fracture healing is less clear as Wnt signaling has both stimulatory and inhibitory effects on osteogenesis. Here, we tested the hypothesis that transient Wnt stimulation promotes the expansion and osteogenesis of a Wnt‐responsive stem cell population present in human bone marrow. Bone marrow mononuclear cells (BMMNCs) were isolated from patients undergoing hip arthroplasty and exposed to Wnt3A protein. The effect of Wnt pathway stimulation was determined by measuring the frequency of stem cells within the BMMNC populations by fluorescence‐activated cell sorting and colony forming unit fibroblast (CFU‐F) assays, before determining their osteogenic capacity in in vitro differentiation experiments. We found that putative skeletal stem cells in BMMNC isolates exhibited elevated Wnt pathway activity compared with the population as whole. Wnt stimulation resulted in an increase in the frequency of skeletal stem cells marked by the STRO‐1^bright^/Glycophorin A^−^ phenotype. Osteogenesis was elevated in stromal cell populations arising from BMMNCs transiently stimulated by Wnt3A protein, but sustained stimulation inhibited osteogenesis in a concentration‐dependent manner. These results demonstrate that Wnt stimulation could be used as a therapeutic approach by transient targeting of stem cell populations during early fracture healing, but that inappropriate stimulation may prevent osteogenesis. Stem Cells *2016;34:418–430*

**Significance Statement**

Modulation of the Wnt pathway may be an attractive therapeutic strategy for bone repair. Wnt stimulation, however, may both inhibit and promote bone differentiation of stem/progenitor cells, depending on when it is applied. Here, we show that putative skeletal stem cells have intrinsically elevated levels of Wnt pathway activation and only a controlled Wnt exposure increases their frequency. We believe this is the first study to specifically address how Wnt stimulation affects putative human skeletal stem cell populations and it provides timely evidence to suggest why novel Wnt therapeutics may be effective in osteoporosis but not in fracture healing.

## Introduction

Bone injury is a major public health problem, the societal and financial implications of which are set to increase rapidly as the average age of our population rises 1, 2. Although bone has a high regenerative capacity, in some cases fracture healing is delayed or absent (resulting in a nonunion) 3. Therapeutic approaches to nonsevere fractures may involve strategies to fix the bones using plates, or the introduction of osteoinductive biomaterials, including allograft or autograft, to stimulate the formation of new bone. Systemic drugs offer the advantage of providing therapy without surgical intervention, but while many systemic drugs acting on a range of targets are clinically approved for stimulating bone formation in osteoporosis (e.g., parathyroid hormone [PTH]), there are little data available to suggest these agents improve fracture healing in humans 4. Therefore, there remains a pressing need for new drugs, targets, and treatment regimens that aim to augment the degree and rate of fracture healing.

One such target is the canonical (β‐catenin‐dependent) Wnt signaling pathway. The importance of Wnt signaling in bone biology is illustrated most strikingly by the corollary of mutations or genetic alterations in components of the canonical Wnt signaling pathway. In humans, genetic studies on high bone mass (HBM) diseases have shown that affected patients often have either *LRP5* gain‐of‐function mutations or *SOST* loss‐of‐function mutations 5, 6, 7. This is also observed in animal models, where mutations that either augment or diminish Wnt signaling result in dramatic bone accrual or loss, respectively 7, 8, 9. Such findings have led to attempts to modulate Wnt signaling for anabolic therapies for osteoporosis or for fracture healing, and there are several therapies presently undergoing clinical trials that target Wnt signaling, including humanized monoclonal antibodies directed to SOST 10 and DKK1 11. These therapies have been developed based on successful pre‐clinical studies which found that these molecules have anabolic effects on bone formation and fracture healing 12, 13, 14. Phase II trials of romosozumab, a humanized monoclonal Ab to SOST, have shown promising results in osteoporosis, and the drug is currently in phase III trials 15, although any positive effect on fracture healing in humans is yet to be proven.

A confounding factor for demonstrating the efficacy of drug modulation of Wnt signaling in fracture healing is the varying requirements for stimulation of this pathway during different phases of fracture healing. For example, Chen et al. found that while selective agonism of the Wnt signaling at late stages of murine fracture healing promoted bone formation, prolonged constitutive activation of β‐catenin resulted in precisely the opposite effect 16. Such in vivo data are reflected in studies on the stem and/or progenitor cells thought to be active in bone healing, marrow stromal cells (MSCs; also commonly referred to as mesenchymal stem cells). In some circumstances, Wnt stimulation inhibits the osteoblastic differentiation of MSCs 17, 18, 19, 20, while in other studies, Wnt stimulation promotes osteogenesis 8, 21, 22, 23. These observations may reflect differing requirements for Wnt stimulation during the lifecourse of an osteoblast—for example, several studies have found that the stimulatory effect of Wnt signaling is dependent on the stage of commitment of the progenitor cell/osteoblast 24, 25, 26. Such data point to a complex situation where Wnt signaling may (a) promote stem/progenitor cell expansion, (b) inhibit early osteoblast differentiation, and/or (c) promote late stage osteoblast differentiation/maturation. A thorough understanding of this situation is further compounded by the lack of agreed or reliable markers for putative stem cells or progenitors that give rise to osteoblasts. In addition, in the majority of published studies, the term “mesenchymal stem cells” refers to isolates of plastic‐adherent stromal cells from bone marrow mononuclear populations 18, 24, 27, 28, 29, 30. Such isolates are also known to contain mixed populations of cells with differing proliferative and differentiation capacities 31, and may themselves contain cells at various stages of commitment. Therefore, a more precise understanding of the effects of Wnt signaling on skeletal stem cells and the progeny at various stages of commitment to the osteogenic lineage is required to determine the optimal time window for therapeutic Wnt stimulation.

In this study, we focused on the effect of Wnt stimulation on fresh isolates of human bone marrow mononuclear cells (BMMNCs) and a population of cells with stem cell‐like properties marked by the STRO‐1^bright^/Glycophorin A (GPA)^−^ cell surface phenotype 32. We tested the hypothesis that putative skeletal stem cell populations in human bone marrow samples are Wnt responsive, and that their commitment to osteogenic differentiation is influenced by Wnt signaling. Furthermore, we determined the effect of Wnt stimulation on cell populations with cell surface marker phenotypes that are known to be enriched in colony forming unit fibroblast (CFU‐F) activity, and measured the effect of either an early transient Wnt stimulation or a prolonged exposure in stromal cells arising from adherent BMMNCs. Our results demonstrate that the timing of Wnt exposure in cells that contribute to fracture healing may be critical to the success of such therapies.

## Materials and Methods

### Reagents

Tissue culture reagents were obtained from Lonza (Basel, Switzerland, www.lonza.com) unless otherwise stated and fetal bovine serum (FBS) from Gibco (Paisley, UK, www. thermofisher.com/uk/en/home/brands/gibco.html). Biochemical reagents were obtained from Sigma‐Aldrich (St. Louis, MO, www.sigmaaldrich.com) unless otherwise stated.

### Cell Extraction and Culture

**Figure 1:**
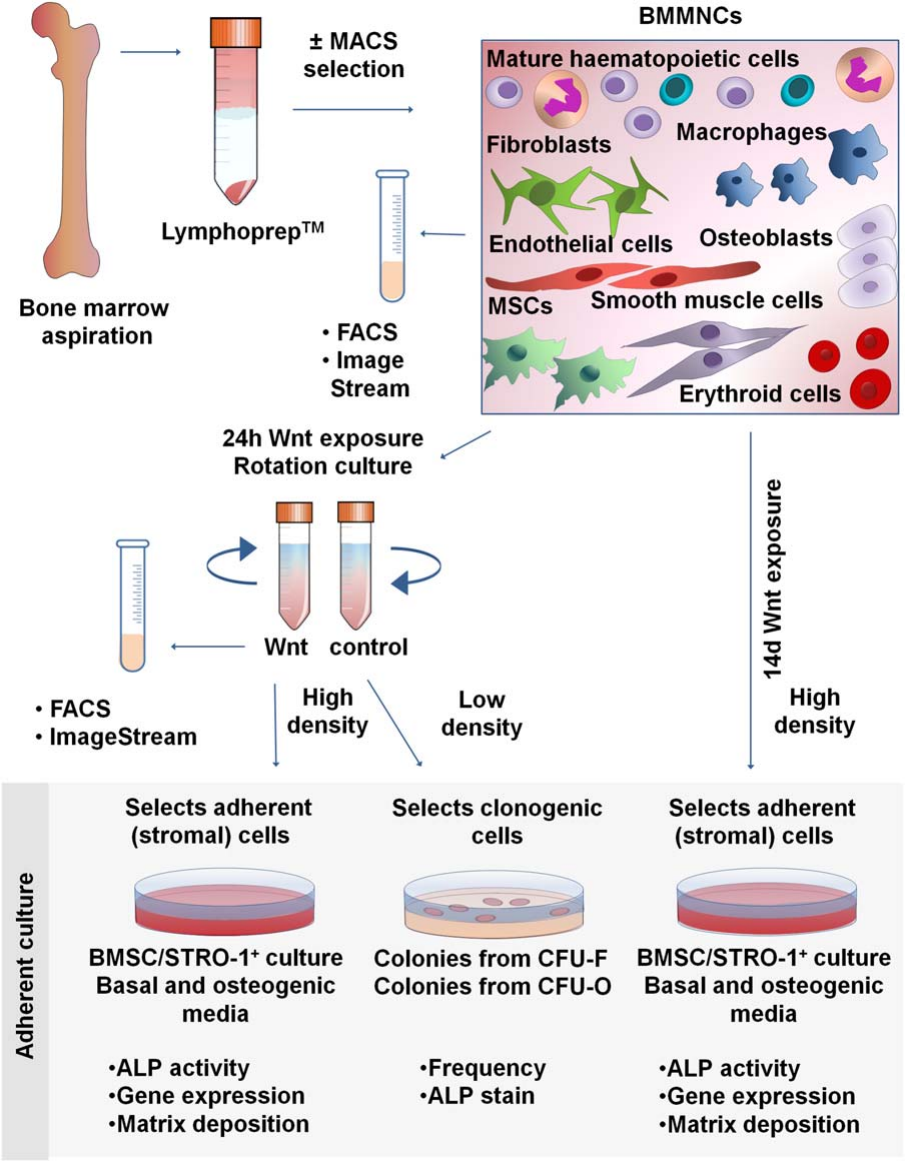
Experimental methodology. Bone marrow mononuclear cells (BMMNCs) were isolated from bone marrow aspirates by Lymphoprep, before further enrichment of a subset of samples by magnetic activated cell sorting selection for the STRO‐1 antigen. Samples were then immediately analyzed by flow cytometry or ImageStream, or were subject to either a transient (24 hours; short‐term) or prolonged (14 days; long‐term) stimulation with Wnt3A. After transient stimulation, cells were analyzed by flow cytometry or ImageStream, or following 14 days of adherent culture in basal or osteogenic media, assayed for osteogenic differentiation potential (high density seeding) or CFU‐F and CFU‐O formation efficiency (low density seeding). Cells were also assayed after 14 days of ongoing, prolonged stimulation with Wnt (high density seeding) for their osteogenic differentiation potential. Abbreviations: ALP, alkaline phosphatase; BMMNCs, bone marrow mononuclear cells; BMSC, bone marrow stromal cell; CFU‐F, colony forming unit fibroblast; CFU‐O, colony formation in the presence of osteogenic medium; FACS, fluorescence‐activated cell sorting; MACS, magnetic activated cell sorting; MSCs, mesenchymal stem cells.

The bone marrow mononuclear cells (BMMNCs) used in this study were isolated from femoral bone marrow samples obtained from hematologically normal individuals undergoing hip replacement surgery at Southampton General Hospital, with the approval of the Southampton Local Research Ethics Committee (LREC 194/99). Primary cultures of human bone marrow stromal cells were established from 11 donors: seven females and four males, 57–94 years of age, mean age 75.1 years, as described previously 33, 34 (see Supporting Information Methods). Cells were maintained in basal medium (α‐MEM containing 10% FBS and 100 μg/ml penicillin/streptomycin) or osteogenic medium (basal medium supplemented with 100 µM ascorbate‐2‐phosphate, 10 nM dexamethasone, and 5 mM β‐glycerophosphate), with the exception of 24 hours exposure to Wnt3A protein (R&D Systems, Minneapolis, MN, www.rndsystems.com), where serum concentration in the media was 5%. Cells were exposed to 100 ng/ml Wnt3A protein, unless otherwise stated, either for 24 hours (short‐term) or 14 days (long‐term). For proliferation and apoptosis experiments, cells were exposed to 100 ng/ml Dkk1 (R&D Systems, Minneapolis, MN, www.rndsystems.com) as an additional control. Media in long‐term cultures were renewed every 2–3 days. All cells were maintained at 37°C in a humidified 5% CO_2_ atmosphere. All studies were conducted using cells from primary (P0) passage culture. A schematic representation of the experimental methodology is shown in Fig. 1.

### Flow Cytometric Analysis and Sorting

Before staining, BMMNCs were treated with EasyLyse Erythrocyte Lysing Reagent (DAKO, Glostrup, Denmark, www.dako.com). For fluorescence‐activated cell sorting (FACS) analysis, 10^6^ to 10^7^ cells were stained and all cells gated as positive showed a fluorescence intensity greater than that detected on 99% of the cells labeled with the isotype‐matched control. Nonspecific antibody binding was blocked with phosphate‐buffered saline (PBS) containing 1% bovine serum albumin (BSA), 10% human serum, and 3% sera from species in which the secondary antibodies were raised. Staining was performed at 4°C for 30 minutes, before washing (with 2 mM EDTA, 0.5% BSA in PBS). Antibodies used are listed in Supporting Information Table 1. For analysis of proliferation, a Click‐iT EdU Alexa Fluor 488 Flow Cytometry Assay was used (Life Technologies, Carlsbad, CA, www.thermofisher.com) and for apoptosis/necrosis detection, Annexin V Apoptosis Detection Kit eFluor 450 (eBioscience, San Diego, CA, www.ebioscience.com). Samples were assayed on a FACS Calibur/Canto II cytometer (BD Biosciences, San Jose, CA, www.bdbiosciences.com) and analyzed using FlowJo v10 software (Ashland, OR, www.flowjo.com).

### ImageStream Cytometry

Data were acquired using a dual‐camera ImageStream X MkII (ISX) equipped with lasers of wavelength of 405 nm (set to 2 mW), 488 nm (set to 100 mW), 642 nm (set to 150 mW), and dedicated 785 nm (SSC, set to 10 mW). Data were collected using a 60 × objective with a numerical aperture of 0.9 and image resolution of approximately 0.33 µm/pixel. Channels and filter information can be found in Supporting Information Table 2A. Data capture was performed with INSPIRE software v.2 (Amnis, Seattle, WA, www.emdmillipore.com), with a minimum of 1.2 × 10^5^ and maximum of 6 × 10^5^ in‐focus (Gradient RMS M01 > 50), nucleated (Intensity Ch07 > 1 × 10^4^) cells captured for each sample, leading to a minimum of 500 cells of the phenotype of interest (CD45^−^/GPA^−^/STRO‐1^+^). Single stained controls were run for each fluorochrome used and a compensation matrix was created to calculate spectral overlap (Supporting Information Table 2B). All image display properties were adjusted linearly on representative cell population images for each channel based on the pixel range of the signal and then applied to the entire data file. A data analysis template was generated from the established settings and selected gates and used for all subsequent analyses.

### Colony Formation and Differentiation Assays

BMMNCs were plated at 5 × 10^3^ to 5 × 10^4^ cells per square centimeter due to high inter‐donor variability in colony forming potential. After 14 days of culture, cells were washed with PBS, fixed with 95% ethanol, and either stained for alkaline phosphatase (ALP) or collected for ALP activity measurements. Staining was conducted using an ALP staining kit (Supporting Information Methods). Absorbance was measured using an ELx800 microplate reader (Biotek, Winooski, Vermont, www.biotek.com) at a wavelength of 415 nm. DNA content was measured using Quant‐iT PicoGreen dsDNA Reagent (Life Technologies, Carlsbad, CA). Fluorescence was measured on an FLx800 fluorescence microplate reader (Biotek, Winooski, Vermont, www.biotek.com) with excitation/emission wavelengths of 480/520 nm. Two percentage of Alizarin Red S solution (pH = 4.2) was used to evaluate calcium deposition in cultured cells and the dye was subsequently extracted with 10% cetylpyridinium chloride solution. Absorbance was measured at 540 nm.

### RNA Extraction and Quantitative Real‐Time PCR

Total RNA was extracted with an RNeasy Mini Kit (Qiagen, Venlo, Netherlands, www.qiagen.com) or PicoPure (Life Technologies, Carlsbad, CA, www.thermofisher.com), for samples with limited cell numbers. Reverse transcription was conducted using the SuperScript VILO cDNA Synthesis Kit (Life Technologies, Carlsbad, CA, www.thermofisher.com). The ΔΔCt method of relative quantification real‐time polymerase chain reaction (PCR) was performed using 7500 Real Time PCR detecting system using primers designed for genes of interest and housekeeping genes (Supporting Information Methods; Table 3).

### Statistical Analysis

Statistical analysis was performed using GraphPad Prism 6 software (GraphPad, La Jolla, CA, www.graphpad.com). Differences in assays between cells exposed to Wnt protein and the control group were analyzed using a *t*‐test or a paired *t*‐test. For nonparametric data, Mann‐Whitney or Wilcoxon's signed rank test was used. The qualitative PCR data were analyzed with the use of Kruskal‐Wallis test with Tukey's post hoc correction. Data are presented as means ± SD and the significance level was set at *p* = 0.05.

## Results

### Putative MSCs Marked by STRO‐1 Have Elevated Markers of Wnt Pathway Activation

**Figure 2:**
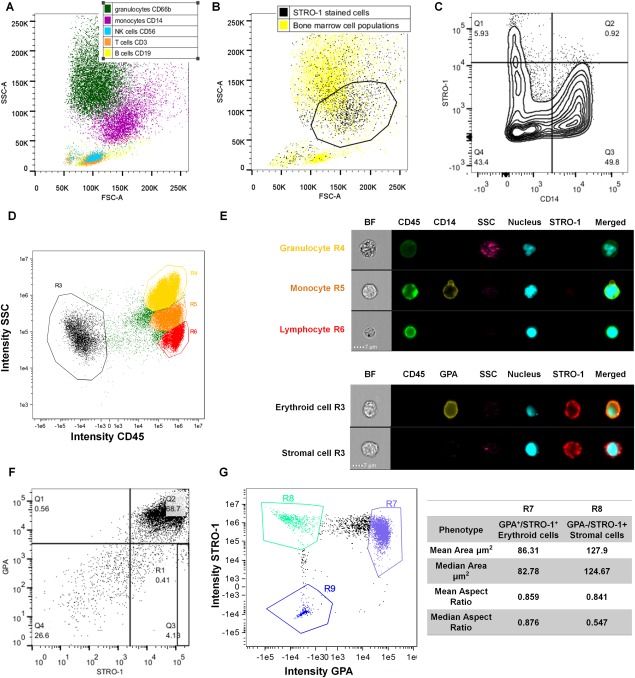
Characterization of putative stromal stem cell populations of the bone marrow. **(A)**: Fluorescence‐activated cell sorting (FACS) dot plot of bone marrow cell populations, backgated according to expression of specific markers. **(B)**: FACS dot plot overlaying STRO‐1‐stained cell population on the top of all bone marrow cell populations, based on FSC versus SSC. Enrichment in the “monocytic” region (marked by black polygon gate) is visible. **(C)**: FACS contour plot depicting that presence of cells double‐positive for STRO‐1 and CD14 (monocyte marker) within the “monocytic” gate is low (Quadrant 2, Q2). **(D)**: Image Stream dot plot and **(E)** panel showing the morphology of CD45^+^ blood populations (granulocytes, monocytes and lymphocytes) and the lack of co‐localization with STRO‐1, and the two STRO‐1^+^ populations within the CD45^−^ fraction of the bone marrow mononuclear cells (erythroid cells, mesenchymal stem cells [MSCs]). **(F)**: FACS dot plot depicting STRO‐1 and Glycophorin A (GPA) co‐localization (Quadrant 2, Q2). The GPA^−^/STRO‐1^+^ fraction was located in quadrant 3 (Q3), and gate R1 demarks GPA^−^/STRO‐1^bright^ population set as top 10% of the GPA^−^/STRO‐1^+^ fraction on the day of bone marrow isolation. G) Dot plot shows the identification of STRO‐1 and GPA expressing cells, and the table shows that GPA^−^/STRO‐1^+^ cells are larger than GPA^+^/STRO‐1^+^ erythroid cells. Abbreviations: BF, brightfield; FSC‐A, forward scatter area; GPA, Glycophorin A; NK, natural killer; SSC‐A, side scatter area.

To confirm the phenotype of STRO‐1^+^ cells, we first performed antibody staining and FACS analysis of freshly isolated BMMNCs. Populations of granulocytes, monocytes, and lymphocytes, including natural killer cells, T cells and B cells, were identified using a panel of antibodies (CD66b, CD14, CD56, CD3, and CD19, respectively) and comprised 65%, 6.63%, 2.93%, 6.37%, and 3.89%, respectively, of the BMMNCs fraction. Labeling corresponded with the known light scattering properties of these cell populations (Fig. 2A). STRO‐1 marked 8.85 ± 4.66% of BMMNCs, and these cells co‐localized based on light scattering properties predominantly with the monocyte population (Fig. 2B; 18.8 ± 8.21%, *n* = 7, of the monocytic fraction based on FSC *vs.* SSC). However, very few STRO‐1^+^ cells also stained with the hematopoietic surface marker CD45 or with the monocyte cell surface marker CD14 (0.88 ± 0.17%, *n* = 3) confirming the STRO‐1 fraction as a distinct subpopulation (Fig. 2C, 2D, 2E), with coincidental similarities in light scattering properties.

While STRO‐1‐selected cells contain all the CFU‐F activity, further enrichment of BMMNCS for cells with stem cell‐like activity has been shown by positive selection for the highest 10% of cells expressing STRO‐1 (STRO‐1^bright^) combined with negative selection for GPA (a marker of erythroid cells) 32. We found that 55.81 ± 10.93% of the recovered cells were STRO‐1^+^/GPA^+^ and that 6.63 ± 2.26% were STRO^+^/GPA^−^, with 0.66 ± 0.22% of the population falling into the STRO‐1^bright^ fraction (Fig. 2F; Supporting Information Fig. 1B). Using ImageStream cytometry, STRO‐1^+^/GPA^−^ could further be distinguished from STRO‐1^+^/GPA^+^ erythroid progenitors using cell morphology, as the STRO‐1^+^/GPA^−^ cells exhibited both significantly greater cell area and lower cell aspect ratio (Fig. 2G; Supporting Information Fig. 1C).

**Figure 3:**
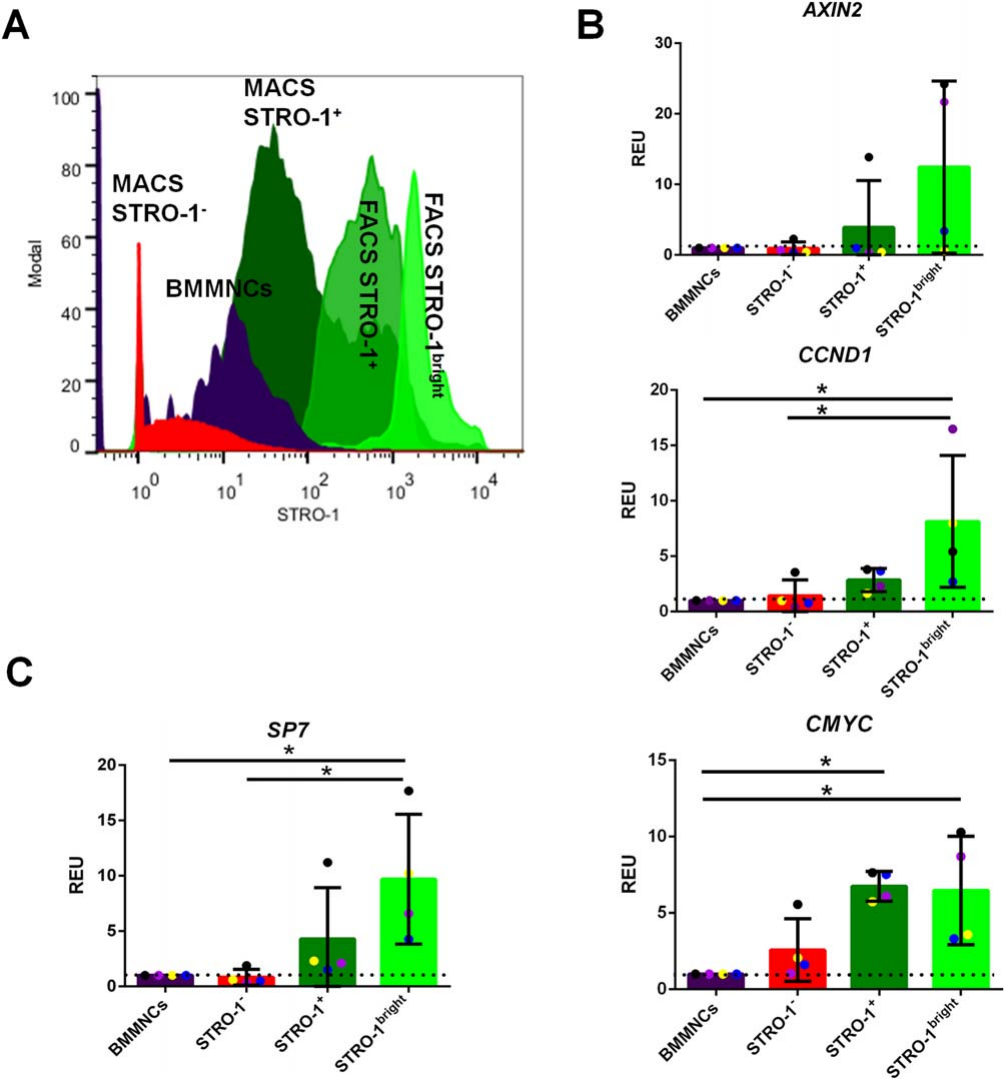
STRO‐1^+^ population within the bone marrow has intrinsically higher Wnt signaling than the general bone marrow population. **(A)**: Fluorescence‐activated cell sorting (FACS) histogram depicting gating for FACS STRO‐1‐sorted samples. The STRO‐1^bright^ cells are the top 10% fraction of the FACS STRO‐1^+^ cells. **(B)**: Qualitative polymerase chain reaction (qPCR) data showing Wnt signaling pathway target genes mRNA expression (*AXIN2*, *CCND1* and *CMYC*, respectively) in FACS‐sorted samples (STRO‐1^−^, STRO‐1^+^, and STRO‐1^bright^ cells as relative to bone marrow mononuclear cells [BMMNCs]), based on the level of STRO‐1^+^ mesenchymal stem cell [MSC] marker expression. All data have been normalized to β*ACTIN* and to 1 for BMMNCs of the same marrow sample. *n* = 4; *, *p* < 0.05. **(C)**: qPCR data showing *SP7* mRNA expression in FACS‐sorted samples (STRO‐1^−^, STRO‐1^+^, and STRO‐1^bright^ cells as relative to BMMNCs), based on the level of STRO‐1^+^ MSC marker expression. All data have been normalized to β*ACTIN* and to 1 for BMMNCs of the same marrow sample. *n* = 4; *, *p* < 0.05. Abbreviations: BMMNCs, bone marrow mononuclear cells; FACS, fluorescence‐activated cell sorting; MACS, magnetic activated cell sorting; REU, relative expression units.

As STRO‐1^+^ cells contain a multipotent population of stem cells, and considering there are data suggesting that Wnt may control the self‐renewal of such cells, we reasoned that Wnt signaling may be elevated in the putative stem cell‐enriched populations marked by STRO‐1^+^, STRO‐1^+^/GPA^−^, and STRO‐1^bright^. Accordingly, we observed significant increases in the expression of several mRNA markers of Wnt pathway activation, *CCND1* and *CMYC*, in STRO‐1^bright^ cells compared with unsorted BMMNCs (increase in expression of 8.15 ± 5.95 and 6.48 ± 3.55, respectively, *p* < 0.05), and a similar (but nonsignificant) trend observed for *AXIN2* (Fig. 3A, 3B). It has recently been shown that *Osterix/SP7*‐expressing cells can produce Wnts and respond to Wnt signaling to control bone homeostasis 35. Therefore, we tested STRO‐1^+^ cells for the expression of *Osterix/SP7* and found that STRO‐1^bright^ cells expressed higher levels of *Osterix/SP7* than unsorted BMMNCs (9.69 ± 5.86 higher *vs.* control, Fig. 3C). Together, these data demonstrate that isolates of mononuclear cells from human bone marrow aspirates contain a cell population with known stem cell‐like properties with intrinsically elevated levels of Wnt signaling.

### Early Wnt Exposure Expands the Number of STRO‐1^+^ Osteoprogenitors in Bone Marrow Mononuclear Cell Populations

**Figure 4:**
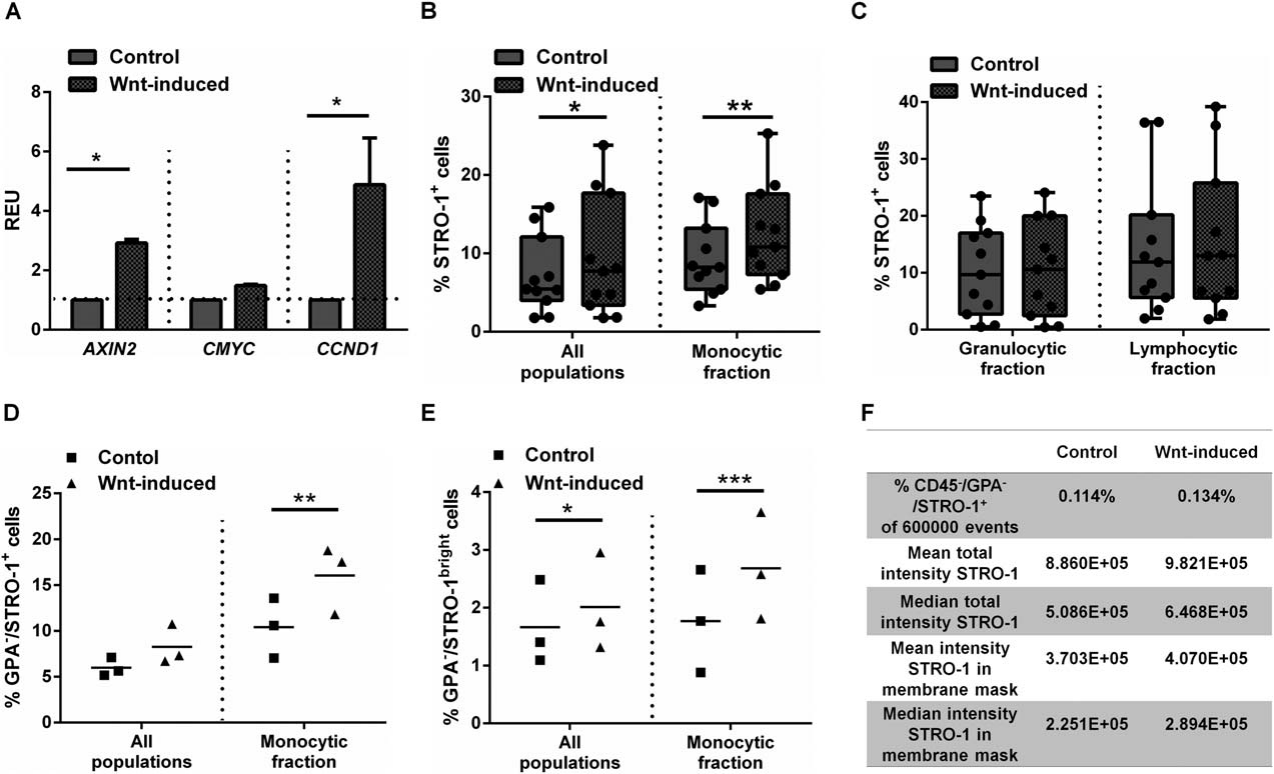
Wnt3A stimulation activates Wnt signaling pathway activity in stromal populations and promotes their expansion. **(A)**: Wnt target gene expression after 24 hours of suspension culture in the STRO‐1‐selected cells. *n* = 3, statistical significance *, *p* < 0.05. **(B)**: STRO‐1 marker expression is augmented within the entire bone marrow and gate co‐localizing with monocytes. (*n* = 11; *, *p* < 0.05; **, *p* < 0.01) or **(C)** unchanged within specific gates co‐localizing with lymphocytes and granulocytes (*n* = 11, ns). The proportion of Glycophorin A (GPA) ^−^/STRO‐1^+^ cells **(D)** or GPA^−^/STRO‐1^bright^ cells **(E)** is increased following Wnt stimulation in the “monocyte” gate (*n* = 3; *, *p* < 0.05; **, *p* < 0.01; ***, *p* < 0.001. **(F)**: Image Stream analysis demonstrates an increase in cell intensity for the STRO‐1 antigen staining following Wnt‐induction in CD45^−^/GPA^−^/STRO‐1^+^ population. Abbreviations: GPA, Glycophorin A; REU, relative expression units.

We next examined whether a transient canonical Wnt stimulus could induce an increase in the frequency of the STRO‐1^+^ population. Isolated BMNNCs were cultured in suspension in the presence of the canonical Wnt protein Wnt3A or a vehicle control for 24 hours, and cell surface expression of STRO‐1 was measured by FACS. We first confirmed Wnt pathway activation by measuring the expression of Wnt target genes *AXIN2, CMYC*, and *CCND1*, in STRO‐1‐selected cells after Wnt3A stimulation. *AXIN2* and *CCND1* expression was significantly increased in Wnt3A‐exposed cells compared with control (by factors of 2.92 ± 0.20 and 4.88 ± 2.72, respectively, *p* < 0.5), with a similar trend for *CMYC* (Fig. 4A).

Following Wnt3A stimulation, we measured an increase in the BMMNC frequency of STRO‐1^+^ cells, with a greater increase measured following gating only for monocytic/stromal fractions (*p* < 0.01; Fig. 4B). There was no Wnt‐dependent increase in STRO‐1 expression in the granulocytic and lymphocytic fractions (Fig. 4C), supporting the notion that the increase in STRO‐1 frequency was confined to cells with scattering properties comparable with monocytes, which include stromal cells. While significant inter‐donor variability in STRO‐1 frequency was observed (ranging from 3.32 to 17.10% in control *vs.* 5.43 to 25.3% after Wnt treatment), an increase in STRO‐1 frequency was observed in every case (Supporting Information Table 4).

To further test the hypothesis that canonical Wnt stimulation enriches BMMNCs for stem cell‐like populations, the effect of Wnt stimulation on the frequency of STRO‐1^+^/GPA^−^ and STRO‐1^bright^/GPA^−^ cells, which are known to be further enriched for stem cells, was measured. In the three separate donor BMMNCs analyzed, we measured an increase in the frequency of either STRO‐1^+^/GPA^−^ cells (by a factor of 1.57 ± 0.16, *p* < 0.01; Fig. 4D) or STRO‐1^bright^/GPA^−^ cells (by a factor of 1.63 ± 0.37%, *p* < 0.001; Fig. 4E). Confirming these measurements, ImageStream analysis not only revealed an increased frequency in the CD45^−^/STRO‐1^+^/GPA^−^ population after Wnt stimulation but also an increased intensity/cell of the STRO‐1 marker expression (Fig. 4F). Together, these data indicate that a short, transient exposure to a canonical Wnt ligand increases the frequency of cells with surface markers that are known to enrich BMMNC populations for stem cells.

**Figure 5:**
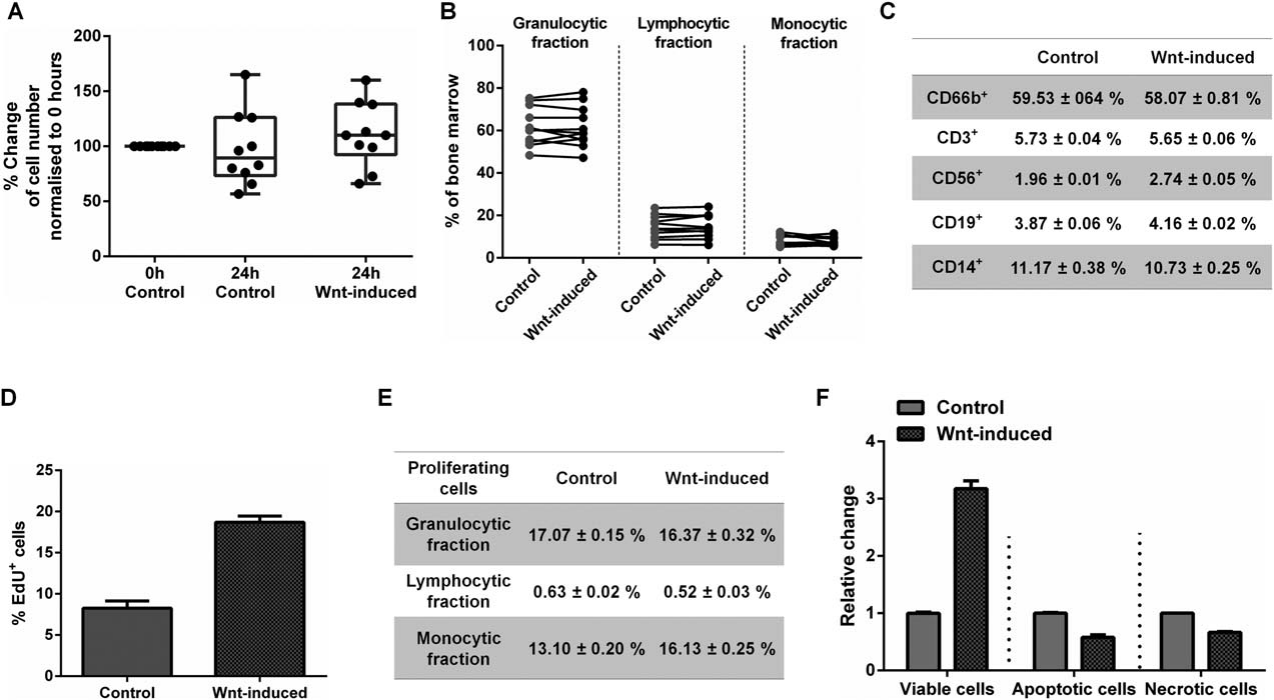
Wnt stimulation increases STRO‐1 proliferation and protects from apoptosis and necrosis. **(A)**: Wnt3A had no effect on the total cell numbers after 24 hours of suspension culture (*n* = 10). **(B)**: Wnt stimulation had no effect on the proportion of the populations of lymphocytes, granulocytes and monocytes in bone marrow mononuclear cells (BMMNC) isolates (*n* = 11). **(C)**: Wnt has no effect on blood cell lineages. Representative figures from triplicates from one patient. **(D)**: Wnt stimulation induces an increase in the frequency of proliferating (EdU^+^) cells within the STRO‐1^+^ population but has no effect on the proportion of EdU^+^ within other populations **(E). (F)**: Wnt stimulation causes an increase in cell viability and a decrease in apoptosis and necrosis (stained with Annexin V and 7‐AAD) within the STRO‐1^+^ population. Data in (D), (E), and (F) presented as mean ± SD, *n* = 1 donor.

We next considered whether canonical Wnt stimulation of mixed BMMNC populations might cause an increase in the frequency of stromal populations by nonspecific effects on other cell populations. To test this, we measured cell number, cell proliferation, cell viability, DNA synthesis, apoptosis, and necrosis in whole populations and subpopulations. Following 24 hours of rotation suspension culture, we found no change in the number of viable cells, as measured by trypan blue exclusion (Fig. 5A). Wnt3A treatment resulted in a small but nonsignificant increase in the total number of cells present after incubation. Furthermore, Wnt3A treatment did not induce changes in the relative numbers of hematopoietic lineages, assessed by measurements of light scattering (Fig. 5B) or by FACS quantification of cells using markers for the hematopoietic lineages (CD3, CD19, CD56, CD14, or CD66a; Fig. 5C). These results indicate Wnt3A effects in the culture system were specific to stromal cell lineages marked by STRO‐1.

We next hypothesized that Wnt3A may exert its effect by providing mitogenic or cell survival signals for stromal populations. To test this, we measured DNA synthesis, an indicator of mitosis and cell proliferation, in Wnt3A or control‐exposed cell populations by EdU incorporation. Apoptotic, necrotic, and viable cells were tracked by Annexin V and 7‐AAD incorporation. A Wnt3A‐dependent increase in 18.70 ± 0.75% *versus* control 8.25 ± 0.89% in the number of proliferating cells in the STRO‐1^+^ population (with stromal cell light scattering properties) was observed (Fig. 5D). There was no evidence of a Wnt3A dependent change in cell proliferation in other cell populations, indicating that the mitogenic effect was specific to stromal populations (Fig. 5E). In addition, we measured an increase in the frequency of viable cells and a decrease in the frequency of apoptotic and necrotic cells in the STRO‐1^+^ subset (Fig. 5F). To try and exclude a possible indirect role for Wnt3A, for example, inducing a paracrine signal in another nonstromal cell population, we attempted to separate populations of STRO‐1^+^ cells to test the effect of Wnt3A exposure in the absence of hematopoietic lineages. In this system, we observed widespread death of STRO‐1 cells (62.78 ± 15.80%, *p* < 0.0001) and were unable to measure any Wnt3A effects (Supporting Information Fig. 2).

It is possible the effects of Wnt3A may be evident on cells expressing other markers ascribed to mesenchymal stem cells, including CD90, CD105, and CD146. However, due to the very low frequency of these cells in BMMNCs, we were unable to measure any effects of Wnt3A on cells expressing markers of putative MSCs (Supporting Information Fig. 3A, 3B for markers in fresh isolates of BMMNCs or Fig. 3C for cultured, plastic adherent stromal cells).

Together, these data indicate that BMMNCs are responsive to stimulus with a canonical Wnt protein, that this induces an increase in frequency in the STRO‐1^+^ population, which is known to contain all CFU‐F activity, and that this is mediated by both protection from apoptosis or necrosis and an increase in cell division, specific to stromal populations.

### Early Wnt Exposure Increases the Frequency of CFU‐Os but Not CFU‐Fs

**Figure 6:**
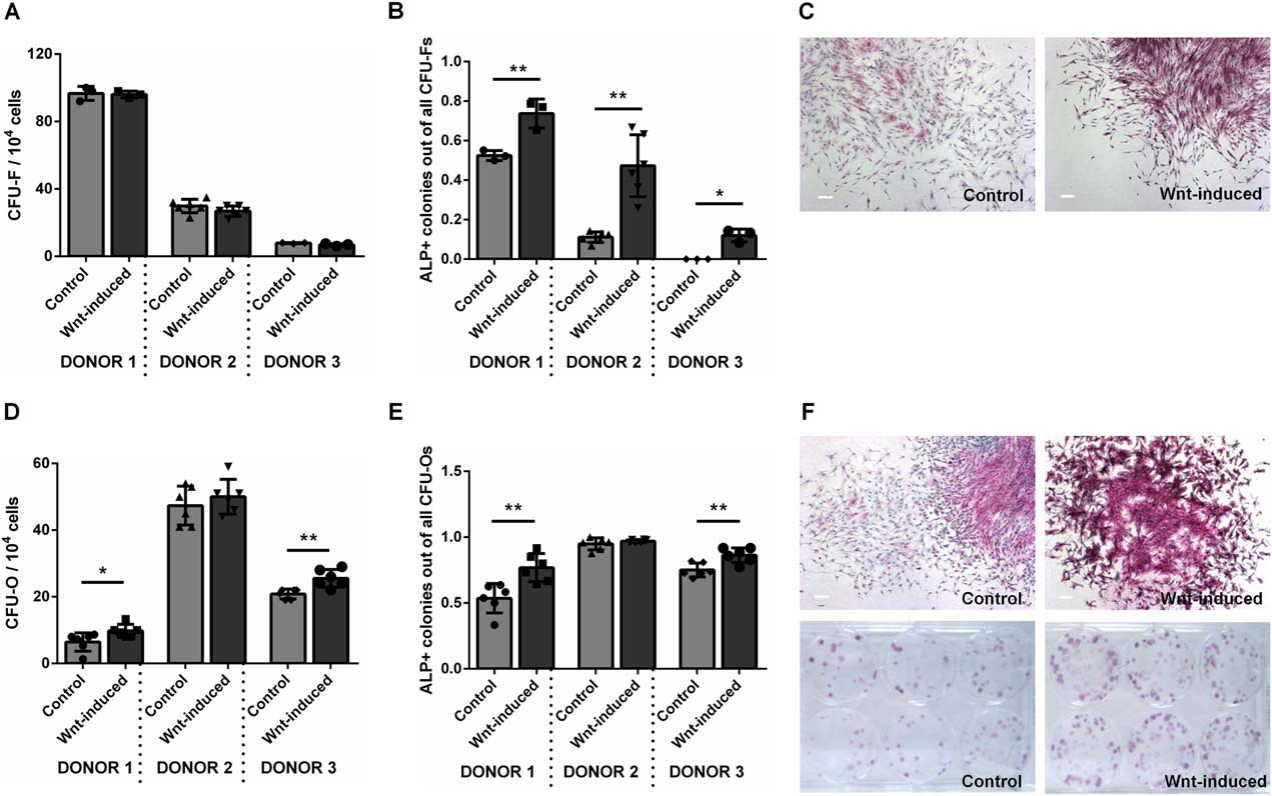
Early Wnt exposure increases the frequency of CFU‐Os but not CFU‐Fs. **(A)** Wnt stimulation of bone marrow mononuclear cells (BMMNCs) has no effect on the frequency of recovered CFU‐F colonies but significantly increases the frequency of those that express ALP **(B). (C)**: ALP staining in CFU‐F colonies arising in basal media. Wnt stimulation of BMMNCs significantly increased the frequency of CFU‐O recovered **(D)** and expression of ALP (E and F) in 2 out of three patients tested *, *p* < 0.05; **, < 0.01. Scale bars = 200 μm. Abbreviations: ALP, alkaline phosphatase; CFU‐F, colony forming unit fibroblast; CFU‐O, colony formation in the presence of osteogenic medium.

Since STRO‐1^+^ cells contain all of the multipotent CFU‐F activity of BMMNCs and are enriched in Wnt‐stimulated BMMNCs, we hypothesized that Wnt stimulation would increase the formation of CFU‐Fs from BMMNC isolates. Surprisingly, we found no increase in the frequency of CFU‐Fs (Fig. 6A). However, on staining colonies for ALP activity, we found that colonies formed from Wnt3A‐stimulated cells had significantly increased ALP activity under basal medium conditions (Fig. 6B, 6C). We considered that this, combined with our measurements of elevated *SP7/*Osterix in previous experiments, was an indicator that the increase in frequency of STRO‐1^+^, STRO‐1^+^/GPA^−^, and STRO‐1^bright^ cells could reflect an increase in the frequency of osteoprogenitors, the growth of which might not be promoted under CFU‐F culture conditions. To test this, we assayed colony formation in the presence of osteogenic medium (CFU‐O assay) and observed a significant increase in the number of CFU‐O recovered (Fig. 6D), and a greater proportion of ALP^+^ CFU‐Os compared with controls (Fig. 6E, 6F). These results suggest that Wnt3A stimulation does not increase the frequency of CFU‐Fs recovered from BMMNC populations, but rather expands a subset of BMMNCs marked by STRO‐1^+^, STRO‐1^+^/GPA^−^, and STRO‐1^bright^ primed for osteogenesis.

### Osteogenic Differentiation in BMMNCs and STRO‐1 Selected Populations Is Promoted by Early Transient Wnt Exposure but Is Abrogated by Prolonged Wnt Stimulation

**Figure 7:**
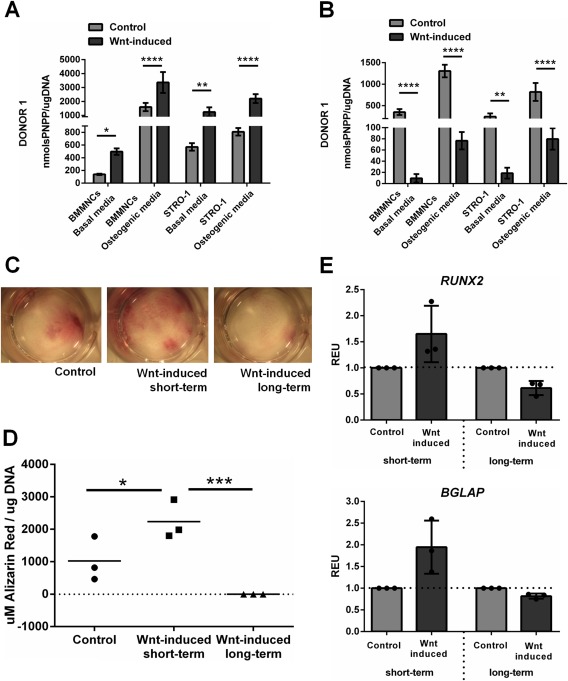
Osteogenic differentiation in bone marrow mononuclear cells (BMMNCs) and STRO‐1‐selected populations is promoted by early transient Wnt exposure but is abrogated by prolonged Wnt stimulation. **(A)**: Transient, early Wnt stimulation causes a significant increase in ALP activity in comparison with control in basal and osteogenic media in general bone marrow cell populations as well as STRO‐1‐selected cells while prolonged Wnt stimulation has the opposite effect **(B)**. This effect is reflected in Alizarin Red staining in cells cultured in osteogenic media **(C)** or by absorbance measurement on the extracted dye **(D)**. Gene expression analysis shows a trend towards an increase in *RUNX2* and *BGLAP* expression after short‐term Wnt induction and decrease after long‐term Wnt induction **(E)**. *, *p* < 0.05; **, *p* < 0.01; ***, *p* < 0.001; ****, *p* < 0.0001. Abbreviations: BMMNCs, bone marrow mononuclear cells; PNPP, p‐nitrophenyl phosphate; REU, relative expression units.

Due to conflicting data in the literature on the effect of canonical Wnt stimulation on the osteogenic differentiation of MSCs 18, 24, 27, we examined whether transient, early Wnt3A stimulation, or a continuous Wnt3A stimulation affected the differentiation of stromal cells arising from BMMNCs. Transient, early stimulation of BMMNCs with Wnt3A resulted in significant increases in the expression of ALP in stromal cell cultures. This was true for both cultures arising from unsorted BMMNCs and cultures from enriched stromal cells expressing STRO‐1^+^. Furthermore, the enhanced expression of ALP occurred regardless of whether culture medium contained osteogenic supplements, although as expected, osteogenic supplementation promoted ALP activity overall (Fig. 7A). Donor‐specific differences in the magnitude of the response to Wnt3A stimulation were observed, but the trend was true for all donors and a significant effect was always measured in osteogenic media (Supporting Information Fig. 4A). In contrast, continuous incubation of stromal cells with Wnt3A, resulted in significant decreases in ALP activity. For example, in BMMNCs cultured in osteogenic media the inhibition reached a mean of 90.86 ± 3.89%, *p* < 0.0001; (Fig. 7B). This indicated a significant reduction in osteogenesis following long‐term Wnt treatment, and was consistent in stromal cells from all donors under all conditions examined (Supporting Information Fig. 4B). This was not due to the concentration employed as a concentration‐dependent inhibition was observed at lower Wnt3A concentrations (25 and 50 ng/ml; Supporting Information Fig. 5). No consistent effects were observed on cell proliferation in adherent cultures of stromal cells, following short‐ and long‐term exposure to Wnt3A (Supporting Information Fig. 6).

To confirm that these ALP activity changes reflected terminal osteogenic differentiation, we performed Alizarin Red S staining to measure matrix mineralization by calcium deposition. Mirroring the data for ALP activity, significantly increased Ca^2+^ deposition in cultures stimulated transiently and decreased Ca^2+^ deposition in cells stimulated long term was observed (Fig. 7C, 7D). No significant differences were observed in the expression of *RUNX2* and *BGLAP* (Osteocalcin), although the trend was similar to the above (Fig. 7E).

These results indicate that transient, early Wnt stimulation augments the osteogenic potential of stromal cells derived from either unsorted or STRO‐1‐sorted BMMNCs, however, sustained Wnt3A activation markedly inhibits osteogenesis.

## Discussion

Modulation of the Wnt signaling pathway is an attractive target for therapies that augment bone formation. Preclinical and clinical trials have indicated that drugs that elevate Wnt signaling can augment bone formation in both animal models and humans, but the target(s) of such drugs remains unresolved 10, 11, 12, 13, 14, 15. This study demonstrates that transient Wnt stimulation of BMMNCs increases the frequency of osteoprogenitors in bone marrow aspirates, but prolonged stimulation abrogates the osteogenic differentiation of the progeny of these cells. Our data show that targeted activation of Wnt signaling in stem or progenitor cells may be critical to therapeutic interventions in fracture healing.

We evaluated the effects of Wnt pathway activation in bone marrow mononuclear cells by using only freshly isolated human bone marrow aspirates, not subjected to passaging or prolonged culture. Such cell sources offer the advantage of the *ex vivo* study of the direct effects of chemical stimulation on cellular milieu found in human bone marrow, rather than on serially passaged, mixed, adherent cell populations. We first investigated a population of cells from the bone marrow, marked by the STRO‐1 antigen, known to contain all of the CFU‐F activity in human bone marrow 36, 37. CFU‐Fs arise from stromal populations in bone marrow isolates, and are indicative of progenitors of the nonhematopoietic lineage including skeletal stem cells, but are likely to contain cell types at different stages of commitment 38. In agreement with other studies, we found the STRO‐1 antibody labeled around 10% of BMMNCs, including cells of the blood lineages, illustrating that the STRO‐1 antigen, while an enrichment marker for skeletal progenitors, is nonspecific for stromal cells in bone marrow 36. Other studies, however, have found that further selection based on the STRO‐1^bright^/GPA^−^ phenotype enriches BMMNCs by three orders of magnitude for CFU‐Fs 32. Cells with this surface phenotype are considered to have stem‐cell‐like properties, exhibiting the ability to differentiate into multiple lineages in vitro and in vivo (cartilage, bone and fat), and to support the formation of hematopoietic marrow when transplanted in vivo 32. While studying their gene expression, we found up‐regulation of several genes involved in Wnt signaling in this population compared with other phenotypes. In addition, we observed Wnt pathway activation and increases in the frequency of these cells following exposure to a canonical Wnt ligand (Wnt3A), which was due to an increase in cell proliferation and reductions in apoptosis and necrosis. Many stem cell populations are known to be regulated by Wnt signaling 39 and although a significant body of published work has focussed on the role of Wnt signaling in cultured marrow stromal cells 18, 20, 22, 24, 28, 29, 40, 41, 42, 43, there are comparatively little data available on the role of Wnt signaling in putative skeletal stem cells, either in their niche in vivo, or in rare populations extracted from the bone marrow 44, 45, 46, 47, 48. Recently, Chan et al. demonstrated that a *de facto* skeletal stem cell population in murine marrow expressed high levels of Wnt ligands and their cognate Frizzled receptors, providing evidence that skeletal stem cell niches may be modulated by Wnts 49. These data are further supported by work on the temporal importance of Wnt signaling in osteoprogenitor cells in development. For example, selective removal of *Wls*, which is necessary for the secretion of Wnts, in cells expressing *Col1a1‐3.6* (a marker of early osteoblastic differentiation) resulted in impairments in the maintenance of MSCs in adult animals 50. Similar effects were measured with the removal of the canonical Wnt ligand Wnt10b 51. In both cases, a decrease in recoverable CFU‐Fs from the bone marrow of these animals was observed, and milder bone phenotypes were observed with the removal of these proteins in more committed osteoblastic cells expressing *Bglap* (*Osteocalcin*). More recently, Tan et al. 35 found that progenitor cells in bone express high levels of Wnt ligands *SP7/Osterix* and *Axin2*, the latter of which is expressed in progenitor cells lining the endosteal surfaces of bone 16. In the data presented here, we found elevated *SP7/Osterix* transcript expression in the STRO‐1^bright^ population, suggesting that this population likely contains similar osteoprogenitor cells.

Despite our observations of an increase in the frequency of STRO‐1^bright^/GPA^−^ cells in human bone marrow after Wnt treatment, we did not measure an increase in the frequency of CFU‐Fs following plating at limiting dilutions, as one might expect if hypothesizing that Wnt stimulation expands a stromal, stem‐like population in our bone marrow aspirates. In contrast, we did observe an increase first in the frequency of CFU‐F colonies that expressed ALP, and second in the frequency of recoverable CFU‐Os. It is possible that Wnt exposure primes uncommitted stromal cells in BMMNC isolates to an osteogenic fate, the clonal growth of which is favored in osteogenic medium. Despite an overall increase in the stromal cell populations, basal cell culture conditions may not be permissive for growth of these expanded cells, hence an increase in their frequency is not evident in CFU‐F assays. This reasoning is supported by our observation of increased osteogenesis of marrow stromal cells derived from Wnt‐stimulated BMMNCs. Taking the data together, it is therefore likely that Wnt exposure “primes” stromal cells in bone marrow aspirates to an osteogenic fate. Notably, continued Wnt exposure dramatically inhibited the osteogenic differentiation of stromal cells, supporting a series of other studies on these cells 18, 19, 20, and indicating that the timing of Wnt exposure is crucial in promoting osteogenic differentiation.

It is important to note that we were unable to access information about the medication status of our human donors, besides their sex and age. One major limitation arising from this might be the inability to assess whether the high inter‐donor variability of the extent of responses to Wnt within the first 24 hours of treatment could be dependent on previous medication. On the other hand, working with samples from patients affected by bone diseases can be advantageous to the clinical relevance of the study, as bone diseases are the underlying cause of failed bone fracture healing, and we noticed positive Wnt effects regardless of the donor disease status. Future studies may seek to stratify the effects of drug intervention based on patient age, disease of medication status.

There is a pressing need to develop therapeutics that augment bone fracture healing, but it is likely that agents that function by anabolic or anti‐catabolic means may fail to prove efficacious in fracture healing without due regard to the complexity of this process. Many drugs including bisphosphonates, PTH/teriparatide, and denosumab are used very successfully in the clinic to augment bone mass and prevent fracture in osteoporosis, but it remains somewhat controversial whether any has clinical benefit in fracture healing 52.

Recent phase II clinical trials on romosozumab have shown positive effects on bone formation and reduced bone resorption 15, and preclinical studies in mice have shown encouraging effects on fracture healing 53, 54. Despite this, the cell type(s) or physiological target(s) affected by SOST inhibition remain unclear. In the case of the former trials, SOST inhibition may act by stimulating osteoblastogenesis, by inhibiting osteoclastogenesis, or by promoting or inhibiting the activity of all cell types within the basic multicellular unit (BMU) of bone, tipping the balance in favor of bone deposition; there is evidence for all these effects 55. The situation in fracture healing is even more complex, where a co‐ordinated series of events over a time period of many weeks ensures bone bridging. Wnt pathway activation may have effects on all these processes, some of which might be stimulatory to healing, and some of which might be inhibitory.

Several of these issues might be solved by a controlled, spatiotemporally defined delivery of drugs to fracture sites. Recently, Swami et al. demonstrated that an approved anticancer drug could be targeted specifically to metastatic bone lesions by the use of nanoparticles modified with bone‐homing surface markers 56. As the bone fracture site is characterized by both an initial hematoma, and a later prolonged period of inflammation, these periods during fracture healing may represent attractive windows when drug targeting might be achieved. Knowledge concerning the times at which cells of the osteoblast lineage are most conducive to stimulation by Wnt signaling, such as data presented in this study, will also inform these approaches.

## Conclusions

In summary, we have demonstrated that transient canonical Wnt stimulation enriches human bone marrow samples for cells expressing known markers of stem/progenitor cells, and leads to a subsequent increase in osteogenesis, which is abrogated if Wnt stimulation is sustained. These results emphasize that therapeutic approaches to modulate Wnt signaling in fracture healing should consider the complexity of Wnt signaling requirements for successful differentiation of osteoblasts from progenitors present at injury sites.

## Author Contributions

A.A.J.: conception and design, collection and/or assembly of data, data analysis and interpretation, manuscript writing, final approval of manuscript; R.S.T. and T.A.N.: conception and design, data analysis and interpretation, final approval of manuscript; E.S. and D.J.: collection and/or assembly of data, data analysis and interpretation, final approval of manuscript; I.M.‐J.: collection and/or assembly of data; C.R.: data analysis and interpretation, final approval of manuscript; R.O.C.O.: conception and design, data analysis and interpretation, financial support, final approval of manuscript; N.D.E.: conception and design, financial support, data analysis and interpretation, manuscript writing, final approval of manuscript.

## Disclosure of Potential Conflicts of Interest

The authors indicate no potential conflicts of interest.

## Supporting information

Supplementary Information
