# Supplementary material for: Transient Canonical Wnt Stimulation Enriches Human Bone Marrow Mononuclear Cell Isolates for Osteoprogenitors
Source: Stem Cells. 2015 Nov 17;34(2):418–30. doi: 10.1002/stem.2241 (PMC4981914; doi:10.1002/stem.2241)
Supplement: Supplementary file 1 — Supplementary Information [file STEM-34-418-s001.pdf]

SUPPLEMENTARY MATERIALS

Supplementary Methods

Establishment of primary cultures of human bone marrow stromal cells

Bone marrow isolates were suspended in plain culture medium (α-MEM), centrifuged to remove fat and strained through a 22µm mesh to remove bone fragments, before gradient separation *via* Lymphoprep^{TM} (Axis-Shield, Oslo, Norway). The BMMNC fraction was collected and either used for the experiments or further enriched for putative MSC populations (5 donors) with the use of STRO-1 antibody (produced by a hybridoma cultured "in house") and magnetic cell separation. This was done by incubation with blocking buffer (PBS containing 1 % BSA, 10 % human serum and 5 % FCS) for 15 min at 4°C, subsequent immunolabelling with neat STRO-1-contianing hybridoma supernatant for 30 min at 4°C and tagging with magnetic bead-conjugated secondary antibody for 30 min at 4°C (130-047-301, Miltenyi Biotec, Bergisch Gladbach, Germany). MACS collection of the STRO-1^{+} fraction was conducted in MACS buffer (2 mM EDTA, 1% BSA in PBS) by using the MidiMACS system and MACS LS columns (Miltenyi Biotec).

Flow cytometric analysis of MSC markers

Before staining, BMMNCs were treated with Easy Lyse Erythrocyte Lysing Reagent (DAKO, Glostrup, Denmark). For FACS analysis 10^{6}x10^{7} cells were stained and all cells gated as positive showed a fluorescence intensity greater than that detected on 99% of the cells labelled with the isotype-matched control. The antibody panel Human Mesenchymal Stem Cell Multi-Color Flow Kit (FMC002) was supplied by R&D Systems (Minneapolis, USA). Samples were assayed on a FACS Canto II cytometer (BD Biosciences, San Jose, USA) and analysed by FlowJo v10 software (FlowJo, Ashland, USA).

ALP quantification

ALP quantification was measured using with Naphthol AS-MX Phosphate and Fast Violet B Salt reagents and counterstaining with Gill no.3 haematoxylin. Colonies which comprised of more than 50 cells with >50 % of area histologically stained in bright purple for ALP were counted as positive. Images were taken on a Zeiss Stemi 2000 dissection microscope with Canon Power Shot G10 digital camera. ALP activity was measured in cells lysed with 0.05% of Triton X-100 with the use of 3mM *p*-nitrophenyl phosphate (PNPP) as substrate, 0.002% octylphenoxypolyethoxyethanol (IGEPAL CA-630) and 1.5M Alkaline Buffer Solution as buffer, as part of Alkaline Phosphatase Activity Kit, and absorbance was read on using an ELx800 microplate reader (Biotek, Vermont, USA) at a wavelength of 415nm

ALP staining quantification vs. cell number

Cells were stained for ALP with a DAPI nuclear stain. For each condition n = 6 pictures was taken of DAPI staining and ALP of random areas within the wells in order to avoid biased selection on a Zeiss Axiovert microscope. The images were analysed using the Cell Profiler 2.1.0. software (MIT), quantifying the intensity of ALP stain in the random area and presenting it in relation to cell count based on number of nuclei within the same area.

Quantitative PCR

Each reaction mixture contained a total of 25 µl with 1 µl of cDNA from the reverse transcription reaction mixture, 5 µM of each primer, and 50 % (vol:vol) of SYBR Green Master Mix (Life Technologies, Carlsbad, USA). The following cycling conditions were used: 50°C for 2 minutes, 95°C for 10 minutes, 40 cycles at 95°C for 15 seconds and 60°C for 1 minute. All reactions were performed in duplicate and product specificity evaluated with the use of melting curves. Specific primer sets were designed for WNT target genes and osteogenic genes and relative gene expression levels were normalized using *βACTIN* housekeeping gene (Supplementary Table 3)

Supplementary Figure and Table Legends

Supplementary Figure 1. A) FACS dot plots of non-selected and of MACS STRO-1-selected bone marrow populations, showing enrichment in the “monocytic” population, and a decrease in “granulocytic” population in the latter. B) Percentages of STRO-1^+, GPA^-/STRO-1^+ and GPA^-/STRO-1^{bright} cells within the entire MACS-selected populations and the monocytic fraction, n = 3. C) Image Stream dot plots depicting Intensity of SSC *vs.* area of cells, showing the GPA^-/STRO-1^+ population has the highest side scatter and area within the $CD45^-$ population of the BMMNCs.

Supplementary Figure 2. Total cell numbers after 24 hours suspension culture shown as % change *vs.* 0 hours; STRO-1-selected samples, n = 6, statistical significance ****p<0.0001 *vs.* 0 hours. Wnt3A had no effect on the total cell numbers after 24 hours of suspension culture on STRO-1-selected samples, n = 6, ns.

Supplementary Figure 3. ***Difficulties in measuring the effect of Wnt with the use of other recognised MSC markers.*** Expression of CD90, CD105 and CD146 in the $CD45^-$ fraction of the entire marrow (A) and gated on monocytes (B) after 24 hour exposure of freshly isolated cells to Wnt. C) Expression of CD90, CD105 and CD146 and CD45 after exposure of cells to Wnt for 24 hours following plastic adherent cell culture.

Supplementary Figure 4. ***Osteogenic differentiation in BMMNCs and STRO-1-selected populations is promoted by early transient Wnt exposure but is abrogated by prolonged Wnt stimulation (additional donors).*** A) ALP activity after short-term Wnt exposure in comparison to control in basal and osteogenic media in general bone marrow cell populations as well as STRO-1-selected cells. Data presented as mean $\pm$ SD; Statistical significance *p<0.05; **p<0.01; **** p<0.0001. B) ALP activity after long-term Wnt exposure in comparison to

control in basal and osteogenic media in general bone marrow cell populations as well as STRO-1-selected cells. Data presented as mean ± SD; statistical significance ***p<0.001; **** p<0.0001.

Supplementary Figure 5. ***Effects of lower doses of long-term Wnt treatment on ALP expression relative to cell number follow a similar trend in abrogating osteogenic differentiation.*** A) ALP intensity relative to cell number for BMMNCs and STRO-1-selected populations cultured in both basal and osteogenic media after a long term 50 ng/ml Wnt treatment and 25 and 100 ng/ml Wnt treatment (B). Separate graphs show data from 3 donors.

Supplementary Figure 6. ***Effect of short- and long-term exposure to Wnt on cell proliferation.*** Cell number for BMMNCs and STRO-1-selected populations cultured in both basal and osteogenic media after short-term Wnt treatment (A) and long term Wnt treatment (B). Statistical significance *p<0.05; **p<0.01; **** p<0.0001, on technical replicates. Separate graphs show data from 3 donors.

Supplementary Table 1.

Antibodies used for phenotyping.

Supplementary Table 2.

ISX Channels and filters and ISX compensation matrix.

Supplementary Table 3.

Primer sequences used for RT-qPCR.

Supplementary Table 4. Detailed donor information (sex, age and disease) and percentages of STRO-1 expressing cells in the entire bone marrow and within the monocytic gate. OA – osteoarthritic; OP – osteoporotic.

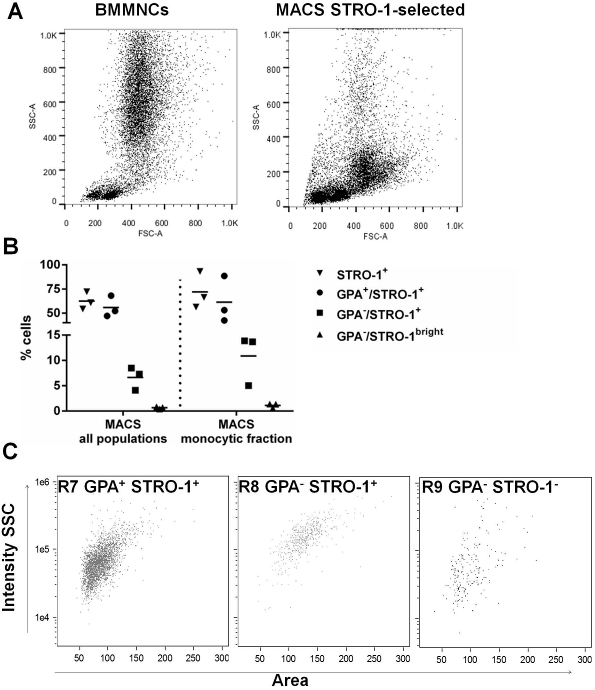

Supplementary Figure 1

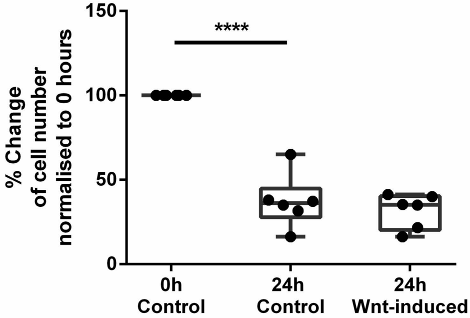

Supplementary Figure 2

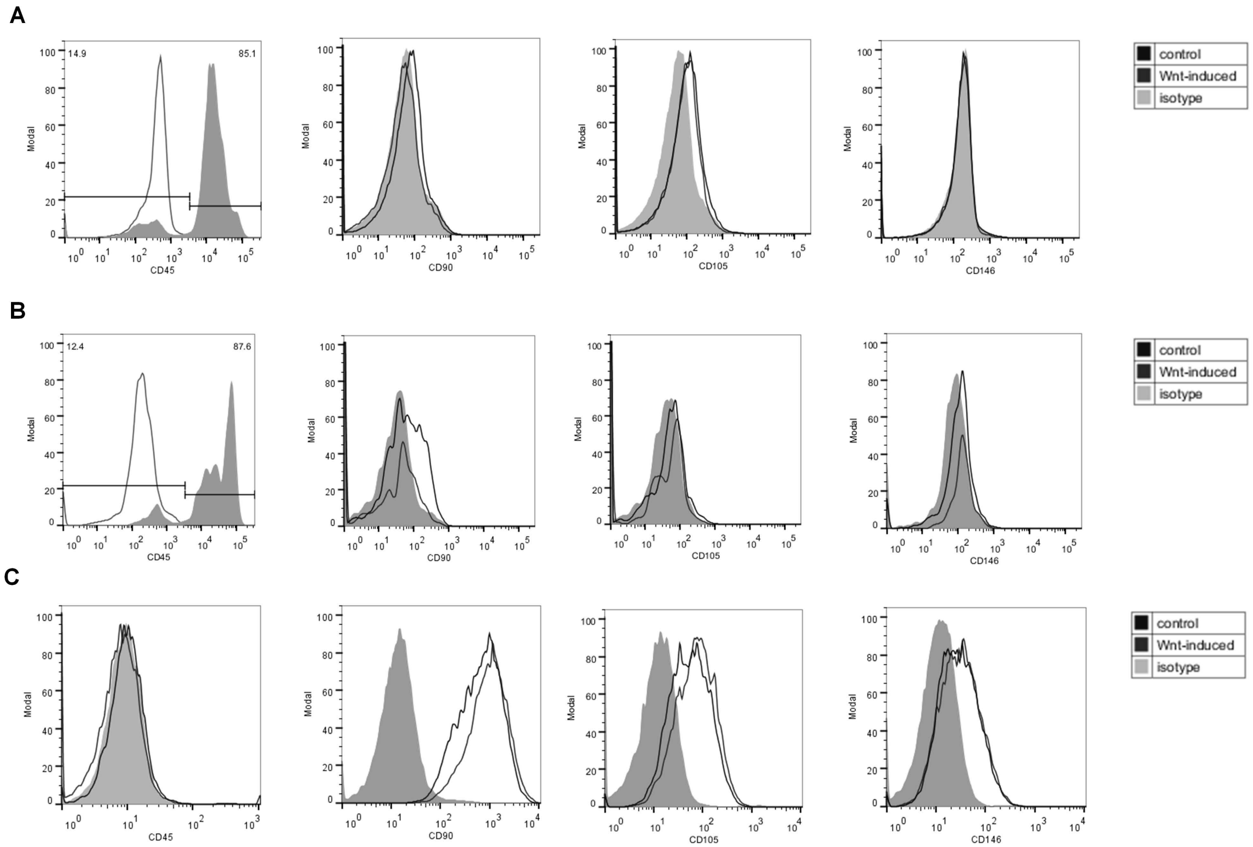

Supplementary Figure 3

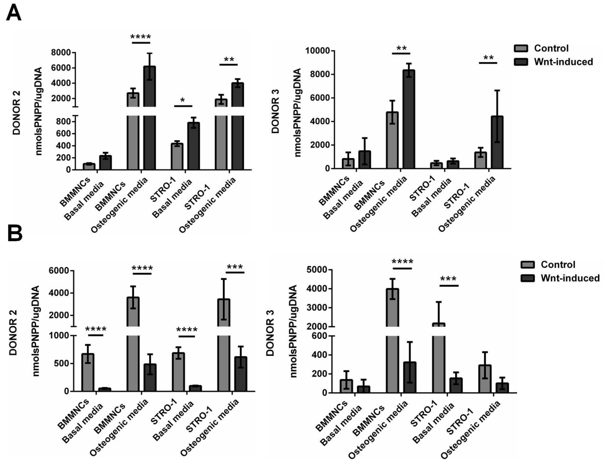

Supplementary Figure 4

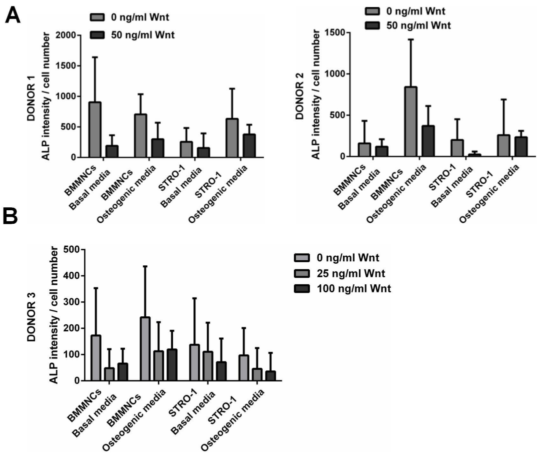

Supplementary Figure 5

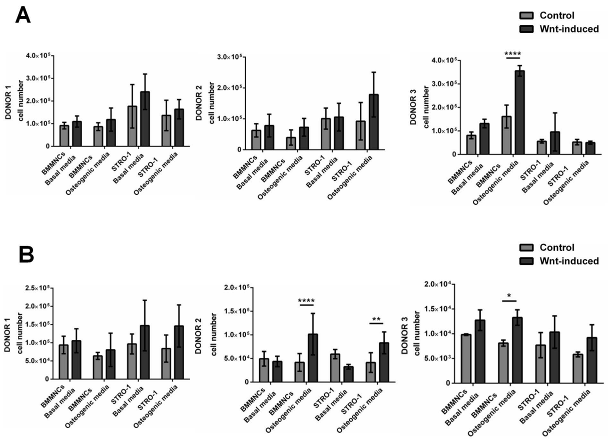

Supplementary Figure 6

**Supplementary Table 1. Antibodies used for phenotyping.**		
**Antibody**	**Catalogue number**	**Supplier**
STRO-1 IgM	MAB1038<br>or made "in house"	R&D Systems, Minneapolis, USA
isotype control IgM	M5909	Life Technologies, Carlsbad, USA
anti-IgM Alexa Fluor 488	A21042	Life Technologies, Carlsbad, USA
anti-IgM Alexa Fluor 647	A21238	Life Technologies, Carlsbad, USA
anti-IgM PE	406507	BioLegend, San Diego, USA
CD19 FITC	made "in house" and kindly supplied by Dr Kam Hussain, Cancer Sciences, University of Southampton	
isotype control IgG1 FITC	400110	BioLegend, San Diego, USA
CD56 PE	318306	BioLegend, San Diego, USA
isotype control IgG1 PE	400112	BioLegend, San Diego, USA
CD3 PerCP	344814	BioLegend, San Diego, USA
isotype control IgG1 PerCP	559425	BD Biosciences, San Jose, USA
CD14 APC	17-0149-41	eBioscience, San Diego, USA
isotype control IgG1 APC	400120	BioLegend, San Diego, USA
CD66b PECy7	25-0666-41	eBioscience, San Diego, USA
isotype control IgG2a PECy7	400231	BioLegend, San Diego, USA
GPA APC	306607	BioLegend, San Diego, USA
isotype control IgG2b APC	400319	BioLegend, San Diego, USA

Supplementary Table 2.

A. ISX Channels and filters.

Channel 1 Camera 1	Channel 2 Camera 1 480-560nm	Channel 3 Camera 1 560-595nm	Channel 6 Camera 1 740-880nm	Channel 7 Camera 2 420-505nm	Channel 9 Camera 2	Channel 11 Camera 2 660-740nm
Brightfield 1	AlexaFluor488	PE	SSC	DAPI	Brightfield 2	AlexaFluor647

B. ISX compensation matrix.

	Ch01	Ch02	Ch03	Ch06	Ch07	Ch09	Ch11
Ch01	1	0.03	0.032	0	0.032	0.001	0.003
Ch02	0.065	1	0.101	0	0.072	0.001	0.028
Ch03	0.006	0.2	1	0	0.037	0.05	0.021
Ch06	0.023	0.025	0.029	1	0.008	0.001	0.008
Ch07	0.03	0.002	0	0	1	0.025	0.033
Ch09	0	0.004	0.12	0	0.195	1	0.028
Ch11	0.001	0.001	0.006	0	0.071	0.021	1

Supplementary Table 3. Primer sequences used for RT-qPCR.				
**Abbreviation**	**Gene and transcript ID**	**Gene name**	**Sequence**	**Amplicon size**
ALP	Gene ID: 249<br>NM_001177520.1	Alkaline phosphatase, liver/bone/kidney	F:5'GGAACTCCTGACCCTTGACC3'<br>R:5'TCCTGTTCAGCTCGTACTGC3'	86bp
AXIN	Gene ID: 8313<br>NM_004655.3	Axin-2; axin-like protein; axis inhibition protein 2; conductin	F:5'CAAGGGCCAGGTCACCAA3'<br>R:5'CCCCCAACCCATCTTCG3'	68bp
βACTIN	Gene ID: 60<br>NM_001101.3	ACTB, actin beta	5'GGCATCCTCACCCTGAAGTA3'<br>5'AGGTGTGGTGCCAGATTTTC3'	82bp
CCND1	Gene ID: 595<br>NM_053056.2	Cyclin D1	F:5'CTACCGCCTCACACGCTT3'<br>R:5'CTTGGGGTCCATGTTCTGC3'	130bp
CMYC	Gene ID: 4609<br>NM_002467.4	MYC v-myc myelocytomatosis viral oncogene homolog; proto-oncogene c-Myc	F:5'CACCACCAGCAGCGACTC3'<br>R:5'GCCTGCCTCTTTTCCACA3'	78bp
OCN	Gene ID: 632<br>NM_199173.4	Osteocalcin; bone gamma-carboxyglutamate protein (BGLAP)	F:5'GGCAGCGAGGTAGTGAAGAG3'<br>R:5'CTCACACACCTCCCTCCT3'	102bp
OSX	Gene ID: 121340<br>NM_001173467.1	Osterix; Sp7 transcription factor	F:5'ATGGGCTCCTTTCACCTG 3'<br>F:5'GGGAAAAGGGAGGGTAATC3'	75bp
RUNX2	Gene ID: 860<br>NM_001024630.3	Runt-related transcription factor 2; CBF-alpha-1	F:5'GTAGATGGACCTCGGGAACC3'<br>F:5'GAGGCGGTCAGAGAACAAAC3'	78bp

Supplementary Table 4. Detailed donor information (sex, age and disease) and percentages of STRO-1 expressing cells in the entire bone marrow and within the monocytic gate.

STRO-1 expression		In the entire bone marrow		In the monocytic fraction	
**No.**	**Sample details**	**Control**	**Wnt-induced**	**Control**	**Wnt-induced**
1	F89 OA	15.90%	23.80%	17.10%	25.30%
2	M91 OA	12.10%	18.70%	16.60%	17.60%
3	M68 OP	5.23%	7.75%	8.24%	10.14%
4	F67 OP	5.46%	4.76%	7.76%	8.49%
5	M84 OP	6.57%	9.29%	7.06%	10.80%
6	F80 OP	14.50%	17.70%	13.20%	18.70%
7	F94 OP	7.07%	8.11%	10.60%	13.10%
8	F57 OP	4.00%	3.40%	5.43%	7.30%
9	M59 OP	5.34%	4.78%	8.42%	13.60%
10	F68 OA	1.85%	1.80%	4.91%	5.91%
11	F69 OA	1.80%	1.85%	3.32%	5.43%

OA – osteoarthritic; OP – osteoporotic.
